# Neuropsychological impairments in emotion recognition compared to general cognition: profiles across six different neurological disorders

**DOI:** 10.1007/s00415-026-13952-5

**Published:** 2026-06-27

**Authors:** Amber Heegers, Sandra E. Rakers, Marieke E. Timmerman, Herma J. Westerhof-Evers, Hugo P. Aben, Esther van den Berg, Nils S. van den Berg, Jay L. P. Fieldhouse, Marleen J. J. Gerritsen, Rob J. M. Groen, Edward H. F. de Haan, Rients B. Huitema, Lize C. Jiskoot, Lieke S. Jorna, Sara Khosdelazad, Paul L. M. de Kort, Miranda C. A. Kramer, Teus van Laar, Yolande A. L. Pijnenburg, Harro Seelaar, Femke F. Siebenga, Fijanne Strijkert, Hiska L. van der Weide, Sygrid van der Zee, Anne M. Buunk, Jacoba M. Spikman

**Affiliations:** 1 Department of Neurology, Clinical Neuropsychology, University of Groningen, University Medical Center Groningen, Hanzeplein 1, 9713 GZ Groningen, The Netherlands; 2 Department of Psychometrics and Statistics, University of Groningen, Groningen, The Netherlands; 3 Department of Neurology, Elisabeth-Tweesteden Hospital, Tilburg, The Netherlands; 4 Department of Neurology and Alzheimer Center Erasmus MC, Erasmus MC – University Medical Center, Rotterdam, The Netherlands; 5 Alzheimer Centre Amsterdam, Department of Neurology, Amsterdam UMC, Amsterdam, The Netherlands; 6 Department of Medical Psychology, Deventer Hospital, Deventer, The Netherlands; 7 Department of Neurosurgery, University of Groningen, University Medical Center Groningen, Groningen, The Netherlands; 8 Donders Institute for Brain, Cognition and Behaviour, Radboud University, Nijmegen, The Netherlands; 9 Department of Radiation Oncology, University of Groningen, University Medical Center Groningen, Groningen, The Netherlands; 10 Department of Neurology, University of Groningen, University Medical Center Groningen, Punt Voor Parkinson, Groningen, The Netherlands; 11 Department of Geriatric Medicine and Alzheimer Center Groningen, University of Groningen, University Medical Center Groningen, Groningen, The Netherlands

**Keywords:** Emotion recognition, Neurocognitive profiles, Traumatic brain injury, Stroke, Subarachnoid haemorrhage, Low-grade glioma, Parkinson’s disease, Frontotemporal dementia

## Abstract

**Objective:**

Social cognition, particularly emotion recognition, can be impaired in neurological disorders involving brain damage and neurocognitive deficits. However, it remains unclear whether distinctive profiles of social versus general cognitive impairments exist across neurological patient groups: moderate–severe traumatic brain injury (mod–sevTBI), acute ischaemic stroke (AIS), aneurysmal subarachnoid haemorrhage (aSAH), frontal low-grade glioma (LGG), advanced Parkinson’s disease (PD), and behavioural variant frontotemporal dementia (bvFTD).

**Methods:**

Data were obtained from scientific studies and clinical records in four Dutch research centres. Neuropsychological testing included emotion recognition [Eckman 60-Faces test (EFT): total score and subscores], memory [Dutch Rey Auditory Verbal Learning Test (DRAVLT): encoding and retrieval], information processing speed, and cognitive control (Trail Making Test A and B). Scores were transformed into Z-scores using normative data and compared across groups.

**Results:**

Included were 710 patients: 118 mod-sevTBI, 93 AIS, 121 aSAH, 100 LGG, 147 PD, 131 bvFTD. EFT-total was impaired in all groups (*p* < .001), with significant group differences (F(5,704) = 30.8, *p* < .001). Emotion recognition was the most severely affected domain in bvFTD, mod-sevTBI, AIS, and LGG. Only bvFTD and mod-sevTBI showed impairments in specific emotions, mainly sadness and fear. MANOVA showed overall group differences in general cognition (Wilks’ Lambda = .69, *p* < .001). Memory encoding was impaired in all groups, but retrieval in none. Information processing speed and cognitive control were impaired only in bvFTD, mod-sevTBI, AIS, and PD.

**Interpretation:**

Emotion recognition is significantly affected across six neurological patient groups, with distinct profiles relative to general cognition. These findings support tailored neuropsychological assessment in clinical practice.

**Supplementary Information:**

The online version contains supplementary material available at 10.1007/s00415-026-13952-5.

## Introduction

Cognitive impairments are prevalent in various neurological disorders characterised by serious brain dysfunction. General cognitive functions, such as memory, processing speed, attention, and executive control, are affected depending on the aetiology, the specific brain regions involved, and the neural networks affected [1–3]. In clinical practice, this knowledge can result in timely neuropsychological assessment, to guide clinical decisions and treatment selection. More recently, social cognition emerged as a crucial neurocognitive domain, but clinical awareness that social–cognitive impairments can be present in these neurological disorders still lags behind [4].

Social cognition encompasses the brain processes enabling appropriate social behaviours and interactions, including the capacities to perceive and interpret social information, understand others, take perspective, and react accordingly [5]. A central aspect of social cognition is emotion recognition; the ability to perceive and recognise another’s emotions, most often based on facial expression. Tests usually address perception of the basic emotions: happiness, anger, disgust, fear, sadness, and surprise. Impairments in emotion recognition are found to detrimentally affect social behaviour, consequently leading to problems in social relationships with a negative impact on societal participation and quality of life [6–10]. Hence, timely identification of such impairments is crucial.

Facial emotion recognition most strongly relies on prefrontal–subcortical networks, including the amygdala and insula [4, 5], which are frequently affected in acute neurological disorders such as moderate–severe TBI (mod–sevTBI), acute ischaemic stroke (AIS), and aneurysmal subarachnoid haemorrhage (aSAH), as well as in more insidious and progressive disorders, such as low-grade gliomas (LGG), Parkinson’s disease (PD), and behavioural variant frontotemporal dementia (bvFTD).

Indeed, impaired overall emotion recognition has been demonstrated in each of these patient groups [8, 11–15]. However, the extent to which the severity of these impairments is comparable across groups remained unclear. Meta-analyses suggest that deficits are particularly pronounced in patients with bvFTD, where social–cognitive impairment is a core feature, and in patients with moderate–severe TBI, yet studies enabling direct comparison across these disorders are lacking [11, 16] given that different disease processes differ markedly in onset (acute versus insidious), pathology (focal versus diffuse) localization, and severity, it is plausible that profiles of impairment in recognising specific emotions vary across aetiologies. Prior research indeed suggests distinct patterns across patient groups, although findings are somewhat inconsistent [10–12, 15]. For instance, a review on moderate–severe TBI [11] reported impairments in recognising anger, fear, sadness, and disgust, whereas a more recent meta-analysis [17] identified deficits only in anger and fear. In aSAH, impairments in recognising disgust and anger have been reported [8]. Charbonneau et al. [18] found that AIS patients performed poorly in recognising happiness, surprise, and fear, whilst a more recent study [19] identified impairments only in anger. Similarly, in patients with LGG, Buunk et al. [20] found selective impairment only in recognising anger. For PD, a review by Argaud et al. [15] showed that fear and sadness were most frequently impaired, whereas a recent study in newly diagnosed patients[9] reported deficits in disgust and sadness. In patients with bvFTD, impairments in disgust, fear, and anger were consistently reported [16, 21], yet Jiskoot et. al. [12] found deficits across all emotions. Such discrepancies within aetiologies likely reflect differences in patient group selection, including variation in injury severity or disease stage. Still, the overall picture that arises is that anger, fear, and disgust are most frequently affected across disorders, and happiness the least.

Direct comparisons between well-defined patient groups are, therefore, essential to determine whether impairments in emotion recognition reflect distinct aetiology-specific profiles, in both pattern and severity, or whether they manifest transdiagnostically. Incorporating information about general cognitive impairments is equally important, as this would allow neurocognitive profiles to be characterised with greater precision and enhance their clinical interpretability.

Although studies providing direct cross–disorder comparisons of cognitive profiles are scarce, the existing literature suggests characteristic patterns for specific aetiologies. For instance, slowed information processing speed and impaired attention are long-established hallmarks of severe TBI [22, 23], and these deficits are likely more pronounced in this patient group than in other neurological disorders, although vascular aetiologies, such as AIS and SAH, may also be associated with such impairments. In bvFTD, behavioural and social–cognitive changes are classically considered early features, yet recent work [24] shows that broader cognitive impairments can already be detected in early disease stages. Strijkert et al. [25] compared neurocognitive profiles of patients with bvFTD, Alzheimer’s disease, and vascular dementia, and found that emotion recognition was most severely impaired in bvFTD, memory encoding and retrieval in Alzheimer’s disease, and information processing speed and cognitive control in vascular dementia.

Based on this literature, we hypothesise that the severity of emotion recognition deficits will differ systematically across neurological disorders rather than manifest uniformly. Specifically, impairments are expected to be most pronounced in bvFTD, followed by moderate–severe TBI, whereas disorders with more focal or modality specific cognitive impairments, such as AIS, SAH, LGG, or PD, are expected to show milder or more selective deficits. These patterns suggest the presence of distinct aetiology-specific profiles, a hypothesis that has yet to be tested through direct comparison across well-defined patient groups. Detailed knowledge of social and non-social–cognitive profiles across neurological disorders is clinically important, as it enables timely and targeted assessment, supports appropriate treatment or counselling to reduce the impact on social and societal participation, and may improve opportunities for earlier diagnosis, particularly in bvFTD. Therefore, the aim of the present study was to characterise emotion recognition and neurocognitive profiles across six major neurological disorders in which frontal–subcortical networks are frequently affected (mod-sevTBI, AIS, aSAH, frontal LGG, advanced PD, and bvFTD). Specifically, the study investigates whether distinct patterns and severities of impairment in emotion recognition and general cognition (memory encoding, memory retrieval, speed of information processing, and cognitive control) can be identified across these groups, and to what extent emotion recognition deficits are related to general cognitive impairments within each disorder. Given existing evidence that emotion recognition constitutes a distinct domain that can be selectively impaired, correlations between emotion recognition and other cognitive domains are expected to be low across patient groups.

## Methods

### Study design

This retrospective multicenter cohort study involved collaboration amongst four different hospitals in the Netherlands: University Medical Center Groningen (UMCG), Amsterdam University Medical Center, Erasmus MC University-Medical Center Rotterdam, and Elisabeth-Tweesteden Hospital Tilburg. Data were collected between 2002 and 2022 from previous scientific studies for which ethical approval and participants’ informed consent were already given, and from clinical files, approved by the UMCG Medical Ethical Committee. Inclusion criteria encompassed availability of age at the time of assessment, sex (defined as birth sex according to patients’ electronic medical reports, self-reported gender (options male/female)), educational level (classified according to the Dutch 7-point scale [26]), and raw data of the relevant neuropsychological tests.

### Participants

Included were adult patients (age ≥ 18 years) with six different disease aetiologies (mod-sevTBI, AIS, aSAH, LGG, PD, and bvFTD) for whom cerebral dysfunction affecting social and general cognition was assumed, meeting respective inclusion criteria based on disease severity/stage or lesion location.

#### Moderate-to-severe TBI (mod–sevTBI)

Glasgow Coma Scale (GCS) score of < 13, loss of consciousness (LOC) of ≥ 30 min, Posttraumatic Amnesia (PTA) duration of ≥ 24 h, or mod–sevTBI according to the Mayo Classification System [27]. Data were obtained from the T-ScEmo study [28], (2010–2016, METc2011.094; *n* = 59, mean PTA duration 32.4, SD 41.1 days), and the Executive Treatment study [29] (2002–2008, METc2002/140; *n* = 31, mean PTA duration 20.9, SD 22.3 days). Additional data were retrieved from clinical files in the UMCG (2005–2010, METc2021/400; *n* = 28, Mean GCS 9.5, SD 3.6; mean PTA duration 41, SD 42 days).

#### Acute ischaemic stroke (AIS)

Moderate-to-severe ischaemic stroke with a National Institutes of Health Stroke Scale (NIHSS) score of 5 or higher. Data were obtained from the Procras study [30] (2016–2020, METc NL56378.028.16; *n* = 41, mean NIHSS score 8.5, range 5–22, 39% left hemispheric stroke, 36.5% right hemispheric stroke, 5% bilateral, and 19.5% unidentified) and the FAB4V study [31] (2015–2019, METc 2015.372; *n* = 52, mean NIHSS score 11.6, range: 5–25, 40.4% left hemispheric stroke, 46.2% right hemispheric stroke, 7.7% bilateral, and 5.7% unidentified).

#### Aneurysmal subarachnoid haemorrhage (aSAH)

Caused by a ruptured intracranial aneurysm (85% of SAH), with availability of the World Federation of Neurological Surgeons (WFNS) grading scale score (range 1–5) [32]. Data were obtained from the SAH study [8] (2010–2017, METc2009/164; *n* = 88 (*n* = 70 WFNS grade 1–3, *n* = 18 WFNS grade 4–5)), and the ICONS study [33] (2018–2022, Netherlands Trial Register NL7803, METc2019/346; *n* = 33 (*n* = 29 WFNS grade 1–3, *n* = 4 WFNS grade 4–5)).

#### Low-grade gliomas (LGG)

Frontally located LGG including diffuse oligodendrogliomas (IDH-mutated, 1p/19q codeleted) and astrocytomas (IDH-mutated, WHO grades 2 and 3). Data of LGG patients assessed after surgery but before adjuvant treatment (radiotherapy, chemotherapy) were retrieved from the clinical database in the UMCG (2014–2021, METc2021/400).

#### Parkinson’s disease (PD)

Advanced stage of PD was evidenced by eligibility to undergo deep brain stimulation (DBS) [34]. Data from patients referred for DBS were retrieved from the clinical database of the UMCG (2010–2021, METc2021/400; mean illness duration 10.1 years, range: 2–34, mean scores UPDRS III OFF medication [35]: 38.7 (SD: 12.8), range: 11–71, mean Hoehn & Yahr score: 2.3 (SD 0.5), range: 1–5).

#### Behavioural variant frontotemporal dementia (bvFTD)

According to the Rascovsky criteria [36], data were collected from clinical files (2012–2020) of the memory clinics in Alzheimer Center Rotterdam Erasmus MC (*n* = 39) (METc 2009–409; 2016–069), Alzheimer Center Amsterdam (*n* = 78), and Alzheimer Center Groningen (*n* = 14) (METcUMCG 2021/400).

### Procedures and measures

Test assessments followed the standard administration procedures, which have remained consistent over time. All raw test scores were transformed into percentile scores using recent norm data of healthy control groups, correcting each individual performance for age, sex, and educational level, eliminating systematic group differences related to these factors. Then, percentile scores were converted to normalised Z-scores. Because of this standardisation, the mean normalised Z-scores of a group on a test can be interpreted as a standardised effect size measure, expressing the standardised mean difference between healthy controls and the specific group on the test involved.

### Emotion recognition

Facial emotion recognition was investigated using the Ekman 60-Faces Test (EFT) [37]. 60 Pictures of facial expressions of the six basic emotions: happiness, anger, disgust, fear, sadness, and surprise are depicted. Participants must select the correct emotion, with maximum scores of 10 on the separate emotions (EFT-happiness, EFT-anger, EFT-disgust, EFT-sadness, EFT-fear, EFT-surprise) and of 60 on EFT-total. Happiness was not analysed separately in this study, because the norms show very limited variability, with almost 90% of the group obtaining a raw score of 10. Norms were applied for the total score and the individual emotions.

### General cognition

#### Memory; encoding and retrieval

The Dutch Rey Auditory Verbal Learning Test (DRAVLT [38] measures verbal memory, with an immediate recall condition (DRAVLT-IR) in which patients have to encode a list of 15 words, over a total of 5 trials (maximum score 75) and a delayed recall condition (DRAVLT-DR) after a 20-min interval (maximum score 15). Norms were applied for DRAVLT-IR and DRAVLT-DR [39], with, for the latter, an additional correction for DRAVLT-IR.

#### Speed of information processing and cognitive control

The Trail Making Test (TMT) measures speed of information processing in version A (TMTA), in which patients have to connect numbers in ascending order as quickly as possible. Cognitive control is measured in version B (TMTB), in which patients have to connect numbers and letters in an alternating sequence. Time in seconds was noted. Norms were applied for both versions [39].

### Statistical analysis

All analyses were performed in the Statistical Package for Social Sciences (SPSS) version 28 [40], with an overall alpha = 0.05. Patient groups were compared on demographic variables using ANOVA’s and chi-squared tests. Normalised Z-scores (Z-scores for short) for emotion recognition [EFT-total, the EFT-subscores (EFT-anger, EFT-disgust, EFT-fear, EFT-sadness, and EFT-surprise)], verbal memory encoding (DRAVLT-IR) and retrieval (DRAVLT-DR), information processing speed (TMTA), and cognitive control (TMTB) of each patient group were compared against the mean of their healthy normative group (i.e., *Z* = 0) using one-sample t tests, applying Bonferroni corrections to adjust for multiple testing with the Bonferroni corrected alpha set to 0.05/60 = 0.001. To analyse whether the six patient groups show overall group differences in emotion recognition, ANOVA’s were applied to the Z-scores of EFT-total, as well as to each of the EFT-subscores. To analyse whether there are overall group differences in general cognition, MANOVA was applied to the Z-scores of the DRAVLT-IR, DRAVLT-DR, TMTA, and TMTB, followed by univariate ANOVA’s per outcome variable. For all variables, subsequently pairwise comparisons were done using Tukey’s HSD between the different groups, thereby adjusting for multiple testing. Further, within each patient group, differences between the Z-scores of all combinations of the five tests (EFT-total, DRAVLT-IR, DRAVLT-DR, TMTA, TMTB) and of the sub-emotion scores (EFT-anger, EFT-disgust, EFT-fear, EFT-sadness, and EFT-surprise) were analysed using paired sample t tests, with the Bonferroni corrected alpha set to 0.05/10 = 0.005. To investigate whether impairments in different domains co-occurred within groups, Spearman correlations were calculated between Z-scores of EFT-total with DRAVLT-IR, DRAVLT-DR, TMTA, and TMTB, with the Bonferroni corrected alpha set to 0.05/4 = 0.01. 95% confidence intervals were reported, and effect sizes were calculated using Cohen’s d (*d*) and eta squared (*η*^2^). According to the conventional benchmarks, values of Cohen’s d 0.20 indicate a small effect, 0.50 a medium effect, and 0.80 a large effect; values of eta squared of 0.01, 0.06, and 0.14 indicate small-, medium-, and large-effect sizes, respectively. For correlation coefficients (r), values of 0.10–0.30 indicate a low correlation, 0.30–0.50 a medium correlation, and 0.50–1.00 a high correlation (with corresponding negative ranges for negative correlations).

## Results

### Participants

This study included 710 patients across six neurological patient groups: mod-sevTBI (*n* = 118), aSAH, (*n* = 121), AIS (*n* = 93), frontal LGG (*n* = 100), advanced PD (*n* = 147), and bvFTD (*n* = 131). Table 1 shows the demographic characteristics of each patient group. Significant group differences were found for age, sex, and educational level.

**Table 1:** Participant characteristics

	mod–sevTBI	AIS	aSAH	LGG	PD	bvFTD	Group difference	
	(*n* = 118)	(*n* = 93)	(*n* = 121)	(*n* = 100)	(*n* = 147)	(*n* = 131)	F/Χ^2^/H	*p*
Age, years	38.0 (13.7)	64.6 (10.7)	54.1 (10.8)	44.0 (12.2)	61.2 (7.1)	64.5 (9.2)	F = 128.1	< .001
Gender, male	93 (78.8%)	58 (62.4%)	39 (32.2%)	53 (53.0%)	89 (60.5%)	81 (61.8%)	Χ^2^ = 57	< .001
Level of education	5.0 (1.0)	4.7 (1.3)	4.8 (1.1)	5.1 (1.0)	5.0 (1.2)	5.2 (1.1)	H = 12.1	< .05

Data depicted are *n* (%) or mean (SD). mod–sevTBI = moderate-to-severe Traumatic Brain Injury, AIS = Acute Ischaemic Stroke aSAH = aneurysmal Subarachnoid Haemorrhage, LGG = Low-grade glioma, PD = Parkinson’s Disease, bvFTD = behavioural variant of Frontotemporal Dementia, Age = Age at time of measurement. Level of Education (Years of Education (YoE)) = ; 1 = primary school [< 6 YoE], 2 = finished primary school [6 YoE], 3 = did not finish secondary school [7–8 YoE], 4 = finished secondary school [9 YoE], 5 = finished secondary school [10–11 YoE], 6 = finished secondary school [12–16 YoE], and 7 = university degree [> 16 YoE])

### Emotion recognition

#### Comparison to healthy normative group scores

**Fig. 1:**
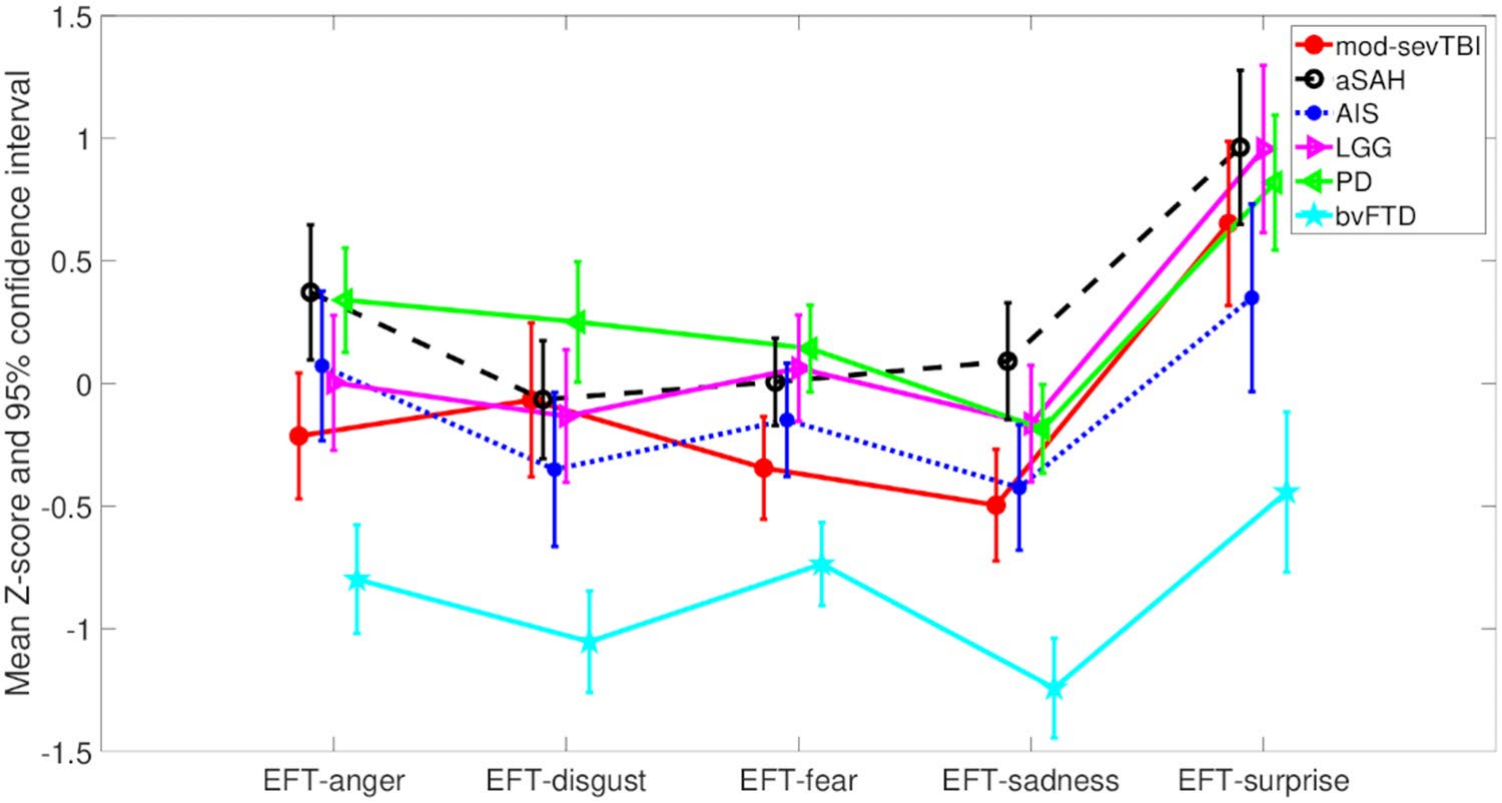
Patterns of separate emotion scores

All patient groups had significantly lower mean EFT-total scores than their healthy normative group (Table 2, Supplementary Table 1, Fig. 1). For the individual emotions, significant differences with the healthy normative groups were found for EFT-sadness (bvFTD and mod–sevTBI), EFT-fear (bvFTD and mod–sevTBI), EFT-anger (bvFTD), and EFT-disgust (bvFTD). For none of the patient groups, recognition of EFT-surprise was impaired. The aSAH, AIS, LGG, and PD groups were not significantly impaired in recognising individual emotions. The impairments in recognising individual emotions differed across patient groups (Fig. 1).

**Table 2:** Comparison of emotion recognition scores (mean(SD)) relative to norm groups and across patient groups

	mod–sevTBI	AIS	aSAH	LGG	PD	bvFTD	Group difference			
	(*n* = 118)	(*n* = 93)	(*n* = 121)	(*n* = 100)	(*n* = 147)	(*n* = 131)	F	*p*	*ŋ*2	[95% CI]
EFT-total										
Percentile score	26.3 (27.7)	26.0 (27.8)	38.5 (29.8)	33.5 (28.9)	39.7 (29.3)	10.0 (16.4)	.	.	.	.
Normalised Z-Score	− .90 (1.1)*	− .90 (1.1)*	− 43 (1.0)*	− .60 (1.1)*	− 37 (1.0)*	− 1.70 (0.8)*	30.8	< .001	.18	[.13, .22]
EFT-anger										
Percentile score	44.4 (33.6)	47.8 (32.5)	54.7 (33.4)	47.9 (31.7)	55.7 (29.1)	29.1 (29.9)	.	.	.	.
Normalised Z-Score	− .19 (1.4)	.08 (1.5)	.35 (1.5)	.00 (1.4)	.33 (1.3)	− .80 (1.2)*	12.7	< .001	.08	[.04, .12]
EFT-disgust										
Percentile score	43.5 (34.7)	35.0 (32.0)	43.4 (29.9)	42.1 (30.5)	50.3 (31.8)	21.4 (24.8)	.	.	.	.
Normalised Z-Score	− .09 (1.6)	− .40 (1.4)	− .09 (1.3)	− 14 (1.4)	.25 (1.5)	− 1.00 (1.2)*	13.0	< .001	.09	[.05, .12]
EFT-fear										
Percentile score	39.6 (30.4)	43.2 (30.7)	50.0 (28.4)	47.7 (29.1)	53.4 (29.5)	28.0 (24.8)	.	.	.	.
Normalised Z-Score	− .35 (1.1)*	− 21 (1.1)	.01 (1.0)	.05 (1.1)	.16 (1.1)	− .76 (0.9)*	13.6	< .001	.09	[.05, .12]
EFT-sadness										
Percentile score	37.7 (30.9)	39.7 (31.7)	50.4 (32.5)	45.1 (31.5)	45.2 (30.4)	19.7 (25.4)	.	.	.	.
Normalised Z-Score	− .50 (1.2)*	− .41 (1.2)	.07 (1.3)	− .17 (1.2)	− .19 (1.1)	− 1.21 (1.2)*	17.5	< .001	.11	[.07, .15]
EFT-surprise										
Percentile score	57.9 (35.2)	49.8 (36.0)	62.8 (34.5)	63.0 (33.8)	61.8 (33.4)	35.1 (35.9)	.	.	.	.
Normalised Z-Score	.64 (1.8)*	.27 (1.8)	.98 (1.7)*	.94 (1.7)*	.81 (1.7)*	− .45 (1.8)	12.1	< .001	.08	[.04, .11]

Data depicted are mean (SD). mod–sevTBI = moderate-to-severe Traumatic Brain Injury, AIS = Acute Ischaemic Stroke, aSAH = aneurysmal Subarachnoid Haemorrhage, LGG = Low-grade glioma, PD = Parkinson’s Disease, bvFTD = behavioural variant of Frontotemporal Dementia

^*^ = Significant difference in comparison to the respective norm group (*with Bonferroni correction, using p* < .001). Test statistics of these comparisons are presented in Supplemententary Table 1

ANOVA post hoc test statistics are presented in Supplementary Table 2

#### Comparison across groups

Comparisons in emotion recognition across groups are presented in Table 2, and post hoc test statistics are presented in Supplementary Table 2. ANOVA showed significant differences between the patient groups on the mean EFT-total, with a large effect size (*ŋ*^*2*^ = 0.18). Post-hoc analyses showed that the bvFTD group scored lower than all other patient groups on EFT-total (*p* <0. ^.^001). The mod–sevTBI group and the AIS group scored lower than aSAH and PD groups on EFT-total (respectively *p* < 0.05 and *p* < 0.001). ANOVA’s showed also for each of the five emotion subscores significant differences between all patient groups, with medium-effect sizes (*ŋ*^*2*^ = 0.08–0.11) (Table 2). Regarding EFT-anger, post hoc analyses showed that the bvFTD group scored lower than all other patient groups (*p* < 0.01) and mod–sevTBI scored lower than the aSAH and PD groups (*p* < 0.05). Concerning EFT-disgust, bvFTD scored lower than all other patient groups (*p* < 0.05), the AIS group scored lower than the PD group (*p* < 0.01). Regarding EFT-fear, the bvFTD group scored lower than all other patient groups (*p* < 0.05), and mod–sevTBI scored lower than the PD group (*p* = < 0.01). Regarding EFT-sadness, the bvFTD group scored lower than all other patient groups (*p* < 0.001), the mod–sevTBI and the AIS groups scored lower than the aSAH group (respectively *p* < 0.01 and *p* < 0.05). Although the recognition of surprise was not impaired in any of the groups, the bvFTD group scored lower on EFT-surprise than all other groups (*p* < 0.05). Table 3 shows a colour-coded pattern of emotion recognition across groups.

**Table 3:**
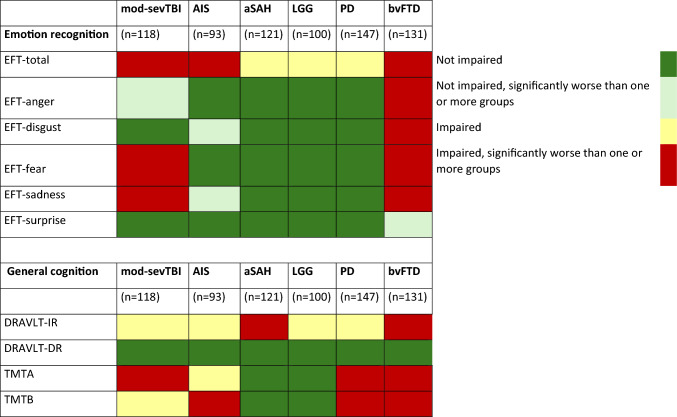
Colour-coded patterns of functioning on emotion recognition and general cognition per patient group, based on mean Z-scores

### General cognition

#### Comparison to healthy normative group scores

**Fig. 2:**
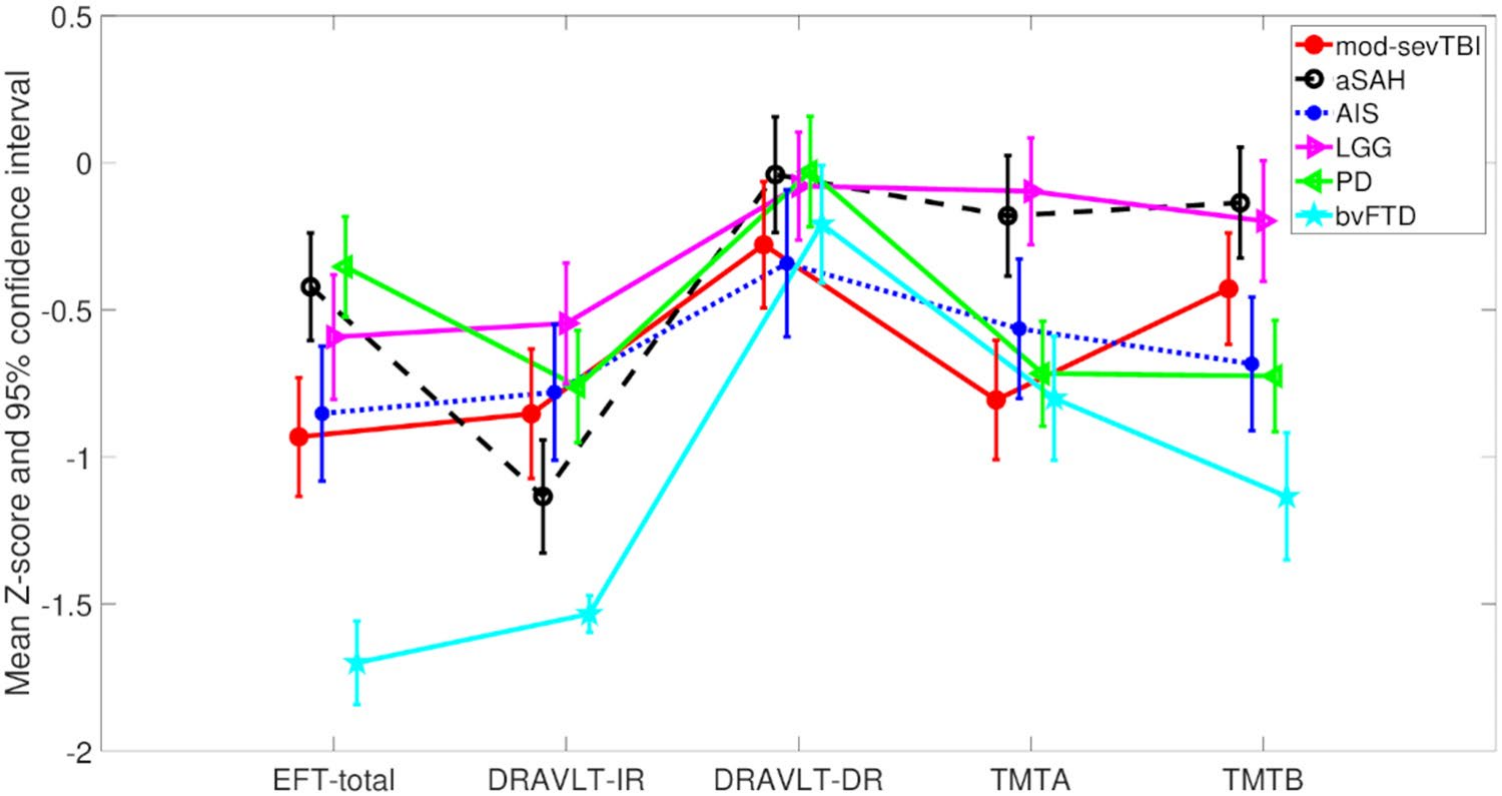
Patterns of emotion recognition total scores and general cognition scores

All patient groups had significantly lower mean DRAVLT-IR scores than their healthy normative group, but no significant impairments were found for the mean DRAVLT-DR scores (Table 4, Supplementary Table 1, Fig. 2). The patients with mod-sevTBI, AIS, PD, and bvFTD performed significantly lower on both TMTA and TMTB than their healthy normative group.

**Table 4:** Comparison of general cognition scores (mean(SD)) relative to norm groups and across patient groups

	mod–sevTBI	AIS	aSAH	LGG	PD	bvFTD	Group difference			
	(*n* = 118)	(*n* = 93)	(*n* = 121)	(*n* = 100)	(*n* = 147)	(*n* = 131)	F	*p*	*ŋ*2	[95% CI]
DRAVLT-IR										
Percentile score	29.9 (28.6)	28.7 (27.0)	21.7 (25.7)	35.5 (28.7)	30.2 (29.5)	14.6 (23.2)	.	.	.	.
Normalised Z-Score	− .83 (1.2)*	− .81 (1.1)*	− 1.13 (1.0)*	− .55 (1.1)*	− .79 (1.1)*	− 1.54 (1.0)*	11.4	< .001	.08	[.04, .11]
DRAVLT-DR										
Percentile score	43.5 (31.2)	39.0 (31.0)	51.3 (29.8)	47.8 (27.3)	49.2 (30.9)	43.1 (31.4)	.	.	.	.
Normalised Z-Score	− .27 (1.1)	− .37 (1.2)	− 02 (1.1)	− .10 (.9)	− .03 (1.1)	− .22 (1.1)	1.9	.099	.01	[.00, .03]
TMTA										
Percentile score	29.9 (27.1)	36.8 (30.0)	45.7 (31.5)	47.4 (27.6)	31.2 (29.0)	29.6 (29.8)	.	.	.	.
Normalised Z-Score	− .80 (1.1)*	− .54 (1.1)*	− .19 (1.1)	− .10 (.9)	− .73 (1.1)*	− .80 (1.1)*	9.4	< .001	.07	[.03, .10]
TMTB										
Percentile score	37.5 (27.1)	32.8 (27.6)	46.2 (29.7)	45.0 (29.8)	32.8 (29.5)	23.6 (23.2)	.	.	.	.
Normalised Z-Score	− .43 (1.0)*	− .68 (1.1)*	− .15 (1.0)	− .21 (1.0)	− .72 (1.2)*	− 1.14 (1.2)*	13.3	< .001	.09	[.05, .13]

Data depicted are mean (SD). mod–sevTBI = moderate-to-severe Traumatic Brain Injury, AIS = Acute Ischaemic Stroke aSAH = aneurysmal Subarachnoid Haemorrhage, LGG = Low-grade glioma, PD = Parkinson’s Disease, bvFTD = behavioural variant of Frontotemporal Dementia, DRAVLT-IR = Dutch Rey Auditory Verbal Learning Test immediate recall, DRAVLT-DR = Dutch Rey Auditory Verbal Learning Test delayed recall, TMTA = Trail Making Test A, TMTB = Trail Making Test B

^*^ = Significant difference in comparison to the respective norm group (*with Bonferroni correction, using p* < *.001*). Test statistics of these comparisons are presented in Supplementary Table 1

ANOVA post hoc test statistics are presented in Supplementary Table 3

#### Comparison across groups

Comparisons in general cognition across groups are presented in Table 4, and post hoc test statistics are presented in Supplementary Table 3. MANOVA showed differences in overall general cognition between the groups (Wilks’ Lambda = 0.80, *F*(5,704) = 7.55, *p* < 0.^.^001) with a small-to-medium-effect size (*ŋ*^*2*^ = 0.05). Univariate tests showed that the patient groups significantly differed on DRAVLT-IR, TMTA, and TMTB with medium-to-large-effect sizes (*ŋ*^*2*^ = 0.07–0.09), and that patient groups did not differ significantly on DRAVLT-DR with a low effect size (*ŋ*^*2*^ = 0.01) (Table 4). Regarding DRAVLT-IR, post hoc analyses demonstrated that the bvFTD group scored lower than most other patient groups (*p* < 0.05) except for the aSAH group. The aSAH group scored lower than the LGG group (*p* < 0.01). Regarding TMTA, the bvFTD, mod–sevTBI, and PD groups scored lower than the aSAH and LGG groups *(p* < 0.001). Regarding TMTB, the bvFTD group scored lower than all other groups (*p* < 0.05). The PD and AIS groups scored lower than the aSAH group and the LGG group (*p* < 0.01)*.* Table 3 shows a colour-coded pattern of general cognition and total emotion recognition scores across groups.

### Profiles per patient group

Table 5 shows the ranking of separate emotion scores per patient group and of general cognition test scores per patient group. For all groups, ranking the separate emotion scores showed that EFT-sadness was lowest, except for the aSAH group. For the general cognition and total emotion recognition test scores, mod-sevTBI, AIS, LGG, and bvFTD had their lowest performance on EFT-total. Test statistics, effect sizes, and confidence intervals are presented in Supplementary Table 4 (separate emotion scores) and Supplementary Table 5 (neurocognitive domains).

**Table 5:** Ranking from most impaired to least impaired cognitive test and from most impaired to least impaired emotion for each patient group based on mean z-scores

mod–sevTBI	AIS	aSAH	LGG	PD	bvFTD
(*n* = 118)	(*n* = 93)	(*n* = 121)	(*n* = 100)	(*n* = 147)	(*n* = 131)
Separate emotion scores					
1. EFT-sadness	1. EFT-sadness	1. EFT-disgust	1. EFT-sadness	1. EFT-sadness	1. EFT-sadness
2. EFT-fear	2. EFT-disgust	2. EFT-fear	2. EFT-disgust	2. EFT-fear	2. EFT-disgust
3. EFT-anger	3. EFT-fear	3. EFT-sadness	3. EFT-anger	3. EFT-disgust	3. EFT-anger
4. EFT-disgust	4. EFT-anger	4. EFT-anger	4. EFT-fear	4. EFT-anger	4. EFT-fear
5. EFT-surprise	5. EFT-surprise	5. EFT-surprise	5. EFT-surprise	5. EFT-surprise	5. EFT-surprise
Significant differences within the ranking of EFT-subscores					
1 vs 5 *(p* < .001)	1 vs 4 *(p* < .005)	1 vs 4 *(p* < .005)	1 vs 5 *(p* < .001)	1 vs 2,3,4,5 *(p* < .001)	1 vs 3,4,5 *(p* < .001)
2 vs 5 *(p* < .001)	1 vs 5 *(p* < .005)	1 vs 5 *(p* < .001)	2 vs 5 *(p* < .001)	2 vs 5 *(p* < .001)	2 vs 5 *(p* < .001)
3 vs 5 *(p* < .001)	2 vs 4 (*p* < .005)	2 vs 5 *(p* < .001)	3 vs 5 *(p* < .001)	3 vs 5 *(p* < .001)	.
4 vs 5 *(p* < .001)	2 vs 5 (*p* < .001)	3 vs 5 *(p* < .001)	4 vs 5 *(p* < .001)	4 vs 5 *(p* < .005)	.
.	.	4 vs 5 *(p* < .005)	.	.	.
Emotion recognition total score and general cognition scores					
1. EFT-total	1. EFT-total	1. DRAVLT-IR	1. EFT-total	1. DRAVLT-IR	1. EFT-total
2. DRAVLT-IR	2. DRAVLT-IR	2. EFT-total	2. DRAVLT-IR	2. TMTA	2. DRAVLT-IR
3. TMTA	3. TMTB	3. TMTA	3. TMTB	3. TMTB	3. TMTB
4. TMTB	4. TMTA	4. TMTB	4. TMTA	4. EFT-total	4. TMTA
5. DRAVLT-DR	5. DRAVLT-DR	5. DRAVLT-DR	5. DRAVLT-DR	5. DRAVLT-DR	5. DRAVLT-DR
Significant differences within the ranking of cognitive tests					
1 vs 4,5 *(p* < .001)	1 vs 5 *(p* < .001)	1 vs 2,3,4,5 *(p* < .001)	1 vs 3,4,5 *(p* < .005)	1 vs 4 (*p* < .001)	1 vs 3,4,5 (p < .001)
2 vs 4,5 *(p* < .005)	2 vs 5 (*p* < .001)	2 vs 5 *(p* < .002)	2 vs 4,5 *(p* < .001)	1 vs 5 (p < .001)	2 vs 3,4,5 (p < .001)
3 vs 4 *(p* < .001)	.	.	.	2 vs 4 (*p* < .001)	3 vs 4,5 (p < .001)
3 vs 5 *(p* < .001)	.	.	.	2 vs 5 *(p* < .001)	4 vs 5 (p < .001)
.	.	.	.	3 vs 4 (*p* < .005)	.
.	.	.	.	3 vs 5 *(p* < .001)	.

mod–sevTBI = moderate-to-severe Traumatic Brain Injury, AIS = Acute Ischaemic Stroke aSAH = aneurysmal Subarachnoid Haemorrhage, LGG = Low-grade glioma, PD = Parkinson’s Disease, bvFTD = behavioural variant of Frontotemporal Dementia

Significant difference in comparison to the other patient groups *(with Bonferroni correction, using p* < *.005).* Test statistics, effect sizes, and confidence intervals of these comparisons are presented in Supplementary Tables 4 and 5

### Correlations between emotion recognition and general cognition

For the mod–sevTBI group, EFT-total showed a significant but low correlation with DRAVLT-IR (0.27; *p* < 0.01). In the aSAH group, EFT-total was not significantly correlated with any general cognitive measure. For the LGG group, EFT-total was again significantly but low correlated with DRAVLT-IR (0.26; *p* < 0.01). In the PD group, EFT-total was also significantly but low correlated with DRAVLT-IR (0.27; *p* < 0.01). In the bvFTD group, EFT-total showed significant low-to-moderate correlations with DRAVLT-IR, TMTA, and TMTB (0.27–0.34; all *p* < 0.01).

## Discussion

The current study investigated impairments in emotion recognition and related these to general cognitive impairments in a large cohort consisting of patients with six common neurological brain disorders (mod-sevTBI, AIS, aSAH, advanced PD, frontal LGG, and bvFTD), comparing neurocognitive profiles across patient groups. Consistent with our hypothesis, we found in all six patient groups significantly impaired emotion recognition compared to normative data of healthy individuals, suggesting that in each of these conditions, the prefrontal–subcortical circuits underlying social cognition are affected. However, the severity and pattern of impairments in emotion recognition appeared to differ significantly across patient groups, supporting the notion of distinct aetiology-specific profiles rather than a uniform transdiagnostic deficit. When comparing patient groups, as predicted, overall emotion recognition was most severely affected in patients with bvFTD, followed by the mod-sevTBI but also the AIS group, all performing significantly worse than the other neurological groups. When comparing neurocognitive domains within patient groups, emotion recognition was the most severely affected neurocognitive domain in patients with bvFTD, mod-sevTBI, AIS, and LGG. The ability to perceive other people’s facial emotions and the ability to experience emotions are closely related, as they rely on overlapping neural circuits that enable empathic and socially adequate behaviour [5, 41, 42]. Consequently, impairments in emotion recognition may serve as a marker of increased vulnerability to show socially inappropriate behaviours, which can lead to negative consequences, such as loss of social relationships and reduced societal participation. Several studies have indeed shown that impaired emotion recognition is associated with changes in social behaviour. For instance, in patients with mod–sevTBI, poorer emotion recognition has been linked to proxy-rated difficulties in adhering to social conventions and displaying appropriate social behaviour [7]. In AIS, impaired emotion recognition has been associated with proxy-rated problems in behavioural regulation [19]. In aSAH, deficits were related to apathy and to proxy-rated interpersonal difficulties [8]. In newly diagnosed Parkinson’s disease, lower emotion recognition performance was associated with higher apathy as well as reduced adherence to social conventions [9]. Similarly, in a mixed group of patients with severe TBI and SAH patients, impaired emotion recognition was linked to greater difficulties in showing appropriate social behaviour [43].

Across aetiologies, specific patterns of emotion recognition impairments also emerged. Patients with bvFTD were most severely affected, showing impairments in recognising anger, disgust, fear, and sadness. The mod-sevTBI group showed impairments in fear and sadness. Notably, sadness recognition was most severely impaired in the bvFTD group, and these performances were significantly worse than in all other groups. Additionally, the mod-sevTBI and AIS groups showed poorer performance on the recognition of sadness than the aSAH group. Overall, these three groups performed worse on sadness than on other emotions. Also, in the LGG and PD group, sadness scores were lower than for other emotions. Hence, sadness emerged as the most frequently and most severely affected emotion across disorders, contrary to our expectations based on the previous literature, where fear, anger, and disgust seemed to be the most commonly affected emotions across neurological conditions. Given that adequate perception of sad emotional expressions of others is a prerequisite for empathising with and comforting others, deficits in recognising sadness are likely to contribute to reduced empathy. Indeed, diminished empathy and broader social–behavioural changes are well documented across these patient groups [4], and are a hallmark of bvFTD, often evident early in the disease course [36].

In addition to sadness, fear recognition was specifically impaired in the bvFTD and mod–sevTBI groups. Impaired recognition of fearful expressions is thought to reflect a reduced ability to experience fear, which may increase the likelihood of engaging in behaviours that are risky and possibly dangerous to oneself or others. Several studies have demonstrated such associations: lower fear recognition has been linked to increased risky decision-making in severe TBI [44], in various neurodegenerative diseases, including bvFTD [45], and in patients with an isolated cerebellar stroke [46].

Finally, surprise was not impaired in any of the groups. Also, the AIS, aSAH, LGG, and PD patients were not impaired in the recognition of other individual emotions.

For the general cognition measures, the analyses showed impairments for all patient groups, and also significant differences across the groups for memory encoding, information processing speed, and cognitive control. All groups were impaired in memory encoding, but in none of the groups was memory retrieval, measured relative to memory encoding, affected. Hence, for all patient groups, despite limited encoding of new declarative information in memory, the information that has been encoded is apparently sufficiently retained over time, indicating no disproportional forgetting similar to the episodic memory impairments that are a hallmark of Alzheimer’s disease [25]. Patients with bvFTD, mod–sevTBI, AIS, and PD were all impaired in both information processing speed and cognitive control. Compared to the other groups, speed of information processing was significantly worse in the bvFTD, mod-sevTBI, and PD groups and cognitive control in the bvFTD group. Importantly, impaired emotion recognition was not consistently correlated to impaired general cognition across groups, and if there were significant correlations, these were low and at most moderate. This again corroborates that social cognition must be conceived as a distinctive neurocognitive domain which can be affected by brain damage separately from other neurocognitive domains [47–49].

Collectively, the findings indicate that social–general neurocognition profiles differ across neurological patient groups, with specific profiles characterising each patient group.

As expected, patients with bvFTD were most severely impaired in emotion recognition, but also in all general cognitive domains, except for memory retrieval, compared to the other groups. Although this disorder is generally characterised by social-behavioural changes, our findings are in line with the previous studies in patients with genetic variants of FTD, showing that even in presymptomatic patients, impairments in attention, memory, and executive functions can be present, although these differ across different genetic variants (C9orf72, GRN, MAPT) [24]. The bvFTD group performed on a similar level as the patients with mod-sevTBI and the PD patients on speed of information processing, but significantly worse on encoding unrelated information in memory and cognitive control. These latter two measures tap into higher-order executive capacities and may depend more on prefrontal involvement than the speed of information processing, which may explain why these functions are more severely affected in bvFTD compared to the other groups.

Patients with mod-sevTBI had a largely comparable general cognition–social cognition profile to the patients with AIS, both having the most severe impairment in emotion recognition compared to the other neurocognitive domains, and more severe than patients with aSAH and PD. In the mod–sev TBI group, recognition of sadness and fear were impaired. For patients with mod-sevTBI, impaired emotion recognition has been widely established [11] and related to prefrontally located damage, a frequent consequence of TBI [49]. For patients with ischaemic stroke, impairments in emotion recognition can be found both after left and right hemispheric strokes [13] and were found to be related to damage in frontal areas, as well as the insula [31, 50]. Both groups had impairments in memory encoding. However, as expected, speed of information processing was more affected in the mod–sevTBI group than the AIS group, but the reverse was found for cognitive control.

In patients with advanced PD, memory encoding, information processing speed, and cognitive control were more severely impaired than emotion recognition. None of the sub-emotions were affected in PD. Hence, even though emotion recognition is significantly affected in the PD group, these impairments are not the most prominent compared to impairments in general cognition.

The aSAH and frontal LGG groups had rather similar profiles, but emotion recognition was only in the LGG group the most affected domain. For both patient groups, the previous studies have not found relationships between damage in specific frontal areas in relation to emotion recognition, suggesting that in these groups, more diffuse damage affects the relevant networks [8, 14]. Regarding general cognitive functions, both groups showed only impairment in the encoding of verbal information in memory, and in the aSAH group, this was even more severely affected than in other neurological disorders except bvFTD.

Although the group-level profiles identified in this study provide valuable insight into the typical pattern of cognitive and social–cognitive impairments across different neurological disorders, they are necessarily based on averages. As such, these profiles may serve to raise clinicians’ awareness of the types of impairment that are likely to occur in patients from these diagnostic groups, and may support diagnostic reasoning when symptoms are unclear. However, these profiles cannot be assumed to apply to every individual patient. There was substantial variability within each group. Although we attempted to define patient groups that were as homogeneous as possible in terms of disease severity or stage, it remains likely that differences in severity or disease duration within these groups still influenced test performance. This indicates that not all patients will necessarily show the expected impairment. Therefore, for individual clinical decision-making, it remains essential to conduct comprehensive neuropsychological testing to determine each patient’s specific pattern of strengths and weaknesses. Importantly, such assessments should rely on normscores that adjust for age, sex, and educational level, as was done in the present study, because these demographic factors are known to contribute to individual differences, not only in general cognitive performance, but also in emotion recognition [37].

## Limitations

A main limitation of this study is that patient data were not collected for the purpose of this study, but in different studies, in different time periods, at different locations, and with either a clinical or scientific purpose. However, given that in the Netherlands, medical centres serve limited population sizes and some of the neurological disorders described are relatively rare, it would not have been possible to collect these data in a single centre design within a short time span. We deem our data comparable, because neuropsychological test assessment takes place in a similar, standardised manner across these centres. Moreover, procedures for test assessment and criteria to define specific patient groups have not been changed during the respective time spans in which data were collected. Hence, we have no reason to assume that this assessment in a different time has caused heterogeneity in the data. Regarding the tests available, this was a limited set of generally administered neuropsychological tests, not covering all possible general cognitive domains, nor other possible relevant aspects of social cognition, such as theory of mind (ToM) or empathy. Furthermore, neuroimaging data on brain damage were not consistently available, so unfortunately, no relations could be studied with impaired test performance in any domain. Also, there was no consistent information on possible comorbid psychopathology in the included patient groups, although most of the original studies used psychopathology as an exclusion criterion. Nevertheless, the possibility cannot be ruled out that psychopathology may have contributed to emotion recognition impairments, as several psychiatric disorders are known to affect social cognition. Finally, it was not possible to analyse the available data on happiness recognition, because the normative dataset showed insufficient variability to allow transformation into Z-scores, and the patient scores displayed similarly limited variability. This is unfortunate, as happiness is typically recognised accurately by almost all individuals, meaning that impaired recognition of this emotion might be particularly clinically meaningful.

## Clinical relevance

The current study shows that in these six neurological brain disorders, impaired emotion recognition is a prominent feature, separate from other neurocognitive impairments, and that these disorders are characterised by distinctive profiles of average impairments in social and general cognition. This knowledge may not only advance individual neuropsychological diagnostic procedures but can also contribute to targeted neuropsychological treatments and counselling for patients and their close others.

## Supplementary Information

Below is the link to the electronic supplementary material.

Supplementary file1 (DOCX 123 KB)

## Data Availability

Anonymised data underlying the findings reported in this study are available upon reasonable request from the participating research centres. Due to privacy and ethical considerations, data sharing is subject to compliance with applicable regulations.
