# Supplementary material for: Neuropsychological impairments in emotion recognition compared to general cognition: profiles across six different neurological disorders
Source: J Neurol. 2026 Jun 27;273(7):428. doi: 10.1007/s00415-026-13952-5 (PMC13310198; doi:10.1007/s00415-026-13952-5)
Supplement: Supplementary file 1 — Supplementary file1 (DOCX 123 KB) [file 415_2026_13952_MOESM1_ESM.docx]

|  |  |  |  |  |  |  |  |  |  |  |  |  |  |  |  |  |  |  |  |  |  |  | |  |  |  | |
| --- | --- | --- | --- | --- | --- | --- | --- | --- | --- | --- | --- | --- | --- | --- | --- | --- | --- | --- | --- | --- | --- | --- | --- | --- | --- | --- | --- |
|  | **Supplementary table 1** | | | | | | | | | | | | | | | | | | | | | | | | | | |
|  | Statistics comparisons against normative group (i.e. H_0_: μ = 0) | | | | | | | | | | | | | | | | | | | | | | | | | | |
|  |  | **mod-sevTBI** | | | | **AIS** | | | | **aSAH** | | | | **LGG** | | | | **PD** | | | | | **bvFTD** | | | | |
|  |  | (n=118) | | | | (n=93) | | | | (n=121) | | | | (n=100) | | | | (n=147) | | | | | (n=131) | | | | |
|  |  | t | *p* | *d* | [95% CI] | t | *p* | *d* | [95% CI] | t | *p* | *d* | [95% CI] | t | *p* | *d* | [95% CI] | t | *p* | *d* | [95% CI] | t | | *p* | *d* | [95% CI] |  |
|  | **EFT-anger** | -1.5 | .134 | -.14 | [-.44, .06] | 0.5 | .592 | .06 | [-.22, .38] | 2.6 | .010 | .24 | [.08, .62] | 0.0 | 1 .00 | .00 | [-.27, .27] | 3 .1 | .002 | .29 | [.12, .54] | -7.3 | | <.001* | -.64 | [-1.01, -.58] | |
|  | **EFT-disgust** | -0.6 | .537 | -.06 | [-.39, .21] | -2.7 | .009 | -.28 | [-.69, -.10] | -0.7 | .464 | -.07 | [-.32, .15] | -1.0 | .310 | -.10 | [-.41, .13] | 2 .0 | .042 | .17 | [.01, .49] | -10.3 | | <.001* | -.90 | [-1.24, -.84] | |
|  | **EFT-fear** | -3.4 | <.001* | -.31 | [-.56, -.14] | -1.9 | .057 | -.20 | [-.43, .01] | 0.1 | .466 | .01 | [-.17, .18] | 0.5 | .638 | .05 | [-.16, .27] | 1 .8 | .077 | .15 | [-.02, .34] | -9.5 | | <.001* | -.90 | [-.92, -.60] | |
|  | **EFT-sadness** | -4.5 | <.001* | -.42 | [-.72, -.28] | -3.2 | .002 | -.33 | [-.66, -.15] | 0.6 | .577 | .05 | [-.17, .30] | -1.4 | .157 | -.14 | [-.41, .07] | -2 .1 | .038 | -.17 | [-.37, -.01] | -11.9 | | <.001* | -.83 | [-1.41, -1.01] | |
|  | **EFT-surprise** | 3.9 | <.001* | .36 | [.32, .97] | 1.4 | .154 | .15 | [-.10, .64] | 6 .0 | <.001* | .55 | [.63, 1.25] | 5.5 | <.001* | .55 | [.60, 1.28] | 5 .8 | <.001* | .48 | [.53, 1.1] | -2.9 | | .004 | -.82 | [-.78, -.15] | |
|  | **EFT-total** | -9.5 | <.001* | -.87 | [-1.14, -.74] | -8.2 | <.001* | -.85 | [-1.12, -.68] | -4.7 | <.001* | -.42 | [-.62, -.25] | -5.7 | <.001* | -.57 | [-.81, -.39] | -4.1 | <.001* | -.34 | [-.52, -.18] | -24.3 | | <.001* | -2.13 | [-1.82, -1.55] | |
|  | **DRAVLT-IR** | -7.6 | <.001* | -.71 | [-1.05, -.61] | -7.0 | <.001* | -.74 | [-1.03, -.58] | -11.9 | <.001* | -1.09 | [-1.32, -.94] | -5.3 | <.001* | -.53 | [-.75, -.34] | -8.2 | <.001* | -.68 | [-.98, -.60] | -16.3 | | <.001* | -1.50 | [-1.72, -1.35] | |
|  | **DRAVLT-DR** | -2.5 | .013 | -.24 | [-.48, -.06] | -3.0 | .003 | -.32 | [-.62, -.13] | -0.2 | .829 | -.02 | [-.22, .17] | -0.9 | .394 | -.09 | [-.26, .10] | -0.3 | .766 | -.03 | [-.23, .16] | -2.2 | | .032 | -.20 | [-.42, -.02] | |
|  | **TMTA** | -7.9 | <.001* | -.74 | [-1.00, -.60] | -4.6 | <.001* | -.49 | [-.77, -.31] | -1.8 | .037 | -.17 | [-.39, -.02] | -1.1 | .269 | -.11 | [-.28, .08] | -8.1 | <.001* | -.67 | [-.90, -.55] | -7.6 | | <.001* | -.70 | [-1.01, -.60] | |
|  | **TMTB** | -4.6 | <.001* | -.43 | [-.62, -.25] | -6.0 | <.001* | -.64 | [-.91, -.46] | -1.6 | .055 | -.15 | [-.35, -.04] | -2.1 | .042 | -.21 | [-.42, -.01] | -7.5 | <.001* | -.62 | [-.90, -.53] | -10.5 | | <.001* | -.97 | [-1.01, -.60] | |
|  | \| Data depicted mod-sevTBI=moderate to severe Traumatic Brain Injury, AIS=Acute Ischemic Stroke aSAH=aneurysmal Subarachnoid Haemorrhage, LGG=Low-Grade Glioma, PD=Parkinson’s Disease, bvFTD=behavioural variant of Frontotemporal  Dementia, DRAVLT-IR=Dutch Rey Auditory Verbal Learning Test immediate recall, DRAVLT-DR=Dutch Rey Auditory Verbal Learning Test delayed recall, TMTA=Trail Making Test A, TMTB=Trail Making Test B \| \| --- \| \| * = Significant difference in comparison to the respective norm group (*with Bonferroni correction, using p<.001*). Some statistics indicate lower scores other indicate higher scores than the normative group. \| | | | | | | | | | | | | | | | | | | | | | | | | | | |

|  |  |  |  |  |  |  |  |  | |  |  |  |  | |  |  |  |  | |  |  |  |  | | |  |  | |  | |  | |  | |
| --- | --- | --- | --- | --- | --- | --- | --- | --- | --- | --- | --- | --- | --- | --- | --- | --- | --- | --- | --- | --- | --- | --- | --- | --- | --- | --- | --- | --- | --- | --- | --- | --- | --- | --- |
| **Supplementary table 2** | | | | | | | | | | | | | | | | | | | | | | | |  |  | | |  | |  | |  | |  |
| Statistics post hoc ANOVA | | | | | | | | | | | | | | | | | | | | | | | |  |  | | |  | |  | |  | |  |
| **I** | **mod-sevTBI** | | | | **AIS** | | | | **aSAH** | | | | | **LGG** | | | | | **PD** | | | | |  | **bvFTD** | | | | | | | | |  |
| **EFT-total** | | | | | | | | | | | | | | | | | | | | | | | | | | | | | | | | | |  |
| **II** | MD | SE | *p* | [95% CI] | MD | SE | *p* | [95% CI] | | MD | SE | *p* | [95% CI] | | MD | SE | *p* | [95% CI] | | MD | SE | *p* | [95% CI] | | |  | MD | | SE | | *p* | | [95% CI] | |
| **mod-sevTBI** | … | … | … | … | … | … | … | … | | … | … | … | … | | … | … | … | … | | … | … | … | … | | |  | … | | … | | … | | … | |
| **AIS** | -.04 | .14 | 1.000 | [-.44, .36] | … | … | … | … | | … | ... | … | … | | … | … | … | … | | … | … | … | … | | |  | … | | … | | … | | … | |
| **aSAH** | -.50 | .13 | .002 | [-.88, -.13] | -.47 | .14 | .011 | [-.86, -.07] | | … | ... | … | … | | … | ... | … | … | | … | ... | … | … | | |  | … | | ... | | … | | … | |
| **LGG** | -.34 | .14 | .134 | [-.73, .05] | -.30 | .14 | .305 | [-.71, .11] | | .17 | .14 | .826 | [-.22, .55] | | … | ... | … | … | | … | ... | … | … | | |  | … | | ... | | … | | … | |
| **PD** | -.59 | .12 | <.001 | [-.94, -.23] | -.55 | .13 | <.001 | [-.93, -.17] | | -.08 | .12 | .984 | [-.44, .27] | | -.25 | .13 | .394 | [-.62, .12] | | … | ... | … | … | | |  | … | | ... | | … | | … | |
| **bvFTD** | .75 | .13 | <.001 | [.39, 1.12] | .79 | .14 | <.001 | [.40, 1.18 | | 1.25 | .13 | <.001 | [.89, 1.62] | | 1.09 | .13 | <.001 | [.71, .1.47] | | 1.33 | .12 | <.001 | [.99, 1.68] | | |  | … | | ... | | … | | … | |
| **EFT-anger** | | | | | | | | | | | | | | | | | | | | | | | | | | | | | | | | | |  |
|  | MD | SE | *p* | [95% CI] | MD | SE | *p* | [95% CI] | | MD | SE | *p* | [95% CI] | | MD | SE | *p* | [95% CI] | | MD | SE | *p* | [95% CI] | | |  | MD | | SE | | *p* | | [95% CI] | |
| **mod-sevTBI** | … | … | … | … | … | … | … | … | | … | … | … | … | | … | … | … | … | | … | … | … | … | | |  | … | | … | | … | | … | |
| **AIS** | -.27 | .19 | .710 | [-.81, .27] | … | … | … | … | | … | ... | … | … | | … | … | … | … | | … | … | … | … | | |  | … | | … | | … | | … | |
| **aSAH** | -.54 | .18 | .027 | [-1.05, -.04] | -.27 | .19 | .669 | [-.81, .27] | | … | ... | … | … | | … | ... | … | … | | … | ... | … | … | | |  | … | | ... | | … | | … | |
| **LGG** | -.19 | .19 | .912 | [-.72, .34] | .08 | .20 | .998 | [-.48, .64] | | .35 | .18 | .392 | [-.17, .88] | | … | ... | … | … | | … | ... | … | … | | |  | … | | ... | | … | | … | |
| **PD** | -.52 | .17 | .026 | [-1.00, -.04] | -.25 | .18 | .739 | [-.77, .27] | | .02 | .17 | 1.000 | [-.46, .50] | | -.33 | .18 | .419 | [-.84, .17] | | … | ... | … | … | | |  | … | | ... | | … | | … | |
| **bvFTD** | .61 | .17 | .006 | [.11, 1.10] | .88 | .18 | <.001 | [.35, 1.41] | | 1.15 | .17 | <.001 | [.66, .1.64] | | .80 | .18 | <.001 | [.66, 1.64] | | 1.13 | .16 | <.001 | [.66, 1.60] | | |  | … | | ... | | … | | … | |
| **EFT-disgust** | | | | | | | | | | | | | | | | | | | | | | | | | | | | | | | | | |  |
|  | MD | SE | *p* | [95% CI] | MD | SE | *p* | [95% CI] | | MD | SE | *p* | [95% CI] | | MD | SE | *p* | [95% CI] | | MD | SE | *p* | [95% CI] | | |  | MD | | SE | | *p* | | [95% CI] | |
| **mod-sevTBI** | … | … | … | … | … | … | … | … | | … | … | … | … | | … | … | … | … | | … | … | … | … | | |  | … | | … | | … | | … | |
| **AIS** | .30 | .19 | .626 | [-.25, .86] | … | … | … | … | | … | ... | … | … | | … | … | … | … | | … | … | … | … | | |  | … | | … | | … | | … | |
| **aSAH** | -.01 | .18 | 1.000 | [-.53, .51] | -.31 | .19 | .599 | [-.86, .24] | | … | ... | … | … | | … | ... | … | … | | … | ... | … | … | | |  | … | | ... | | … | | … | |
| **LGG** | .04 | .19 | 1.000 | [-.50, .51] | -.26 | .20 | .795 | [-.84, .32] | | .05 | .19 | 1.000 | [-.49, .59] | | … | ... | … | … | | … | ... | … | … | | |  | … | | ... | | … | | … | |
| **PD** | -.34 | .17 | .354 | [-.84, .15] | -.65 | .19 | .007 | [-1.18, -.12] | | -.34 | .17 | .368 | [-.83, .16] | | -.39 | .18 | .272 | [-.91, .13] | | … | ... | … | … | | |  | … | | ... | | … | | … | |
| **bvFTD** | .95 | .18 | <.001 | [.44, 1.46] | .65 | .19 | .010 | [.10, 1.19] | | .96 | .18 | <.001 | [.45, .1.46] | | .90 | .19 | <.001 | [.37, 1.44] | | 1.29 | .17 | <.001 | [.81, 1.78] | | |  | … | | ... | | … | | … | |
| **EFT-fear** | | | | | | | | | | | | | | | | | | | | | | | | | | | | | | | | | |  |
|  | MD | SE | *p* | [95% CI] | MD | SE | *p* | [95% CI] | | MD | SE | *p* | [95% CI] | | MD | SE | *p* | [95% CI] | | MD | SE | *p* | [95% CI] | | |  | MD | | SE | | *p* | | [95% CI] | |
| **mod-sevTBI** | … | … | … | … | … | … | … | … | | … | … | … | … | | … | … | … | … | | … | … | … | … | | |  | … | | … | | … | | … | |
| **AIS** | -.14 | .14 | .933 | [-.55, .28] | … | … | … | … | | … | ... | … | … | | … | … | … | … | | … | … | … | … | | |  | … | | … | | … | | … | |
| **aSAH** | -.36 | .14 | .085 | [-.75, .03] | -.22 | .14 | .640 | [-.63, .19] | | … | ... | … | … | | … | ... | … | … | | … | ... | … | … | | |  | … | | ... | | … | | … | |
| **LGG** | -.40 | .14 | .053 | [-.81, .00] | -.27 | .15 | .492 | [-.70, .17] | | -.04 | .14 | 1.000 | [-.45, .36] | | … | ... | … | … | | … | ... | … | … | | |  | … | | ... | | … | | … | |
| **PD** | -.51 | .13 | .001 | [-.88,. -.14] | -.37 | .14 | .077 | [-.77, .02] | | -.15 | .13 | .844 | [-.52, .21] | | -.11 | .14 | .967 | [-.50, .28] | | … | ... | … | … | | |  | … | | ... | | … | | … | |
| **bvFTD** | .41 | .13 | .026 | [.03, .79] | .55 | .14 | .002 | [.14, .95] | | .77 | .13 | <.001 | [.39, 1.14] | | .81 | .14 | <.001 | [.42, 1.21] | | .92 | .13 | <.001 | [.56, 1.28] | | |  | … | | ... | | … | | … | |
| **EFT-sadness** | | | | | | | | | | | | | | | | | | | | | | | | | | | | | | | | | |  |
|  | MD | SE | *p* | [95% CI] | MD | SE | *p* | [95% CI] | | MD | SE | *p* | [95% CI] | | MD | SE | *p* | [95% CI] | | MD | SE | *p* | [95% CI] | | |  | MD | | SE | | *P* | | [95% CI] | |
| **mod-sevTBI** | … | … | … | … | … | … | … | … | | … | … | … | … | | … | … | … | … | | … | … | … | … | | |  | … | | … | | … | | … | |
| **AIS** | -.10 | .17 | .992 | [-.57, .38] | … | … | … | … | | … | ... | … | … | | … | … | … | … | | … | … | … | … | | |  | … | | … | | … | | … | |
| **aSAH** | -.57 | .15 | .003 | [-1.01, -.13] | -.47 | .16 | .048 | [-.94, -.00] | | … | ... | … | … | | … | ... | … | … | | … | ... | … | … | | |  | … | | ... | | … | | … | |
| **LGG** | -.33 | .16 | .314 | [-.80,.13] | -.24 | .17 | .744 | [-.73, .26] | | .24 | .16 | .686 | [-.22, .70] | | … | ... | … | … | | … | ... | … | … | | |  | … | | ... | | … | | … | |
| **PD** | -.31 | .15 | .273 | [-.74, .11] | -.22 | .16 | .743 | [-.67, .23] | | .26 | .15 | .505 | [-.16, .67] | | .02 | .15 | 1.000 | [-.42, .46] | | … | ... | … | … | | |  | … | | ... | | … | | … | |
| **bvFTD** | .71 | .15 | <.001 | [.27, 1.14] | .80 | .16 | <.001 | [.34, 1.27] | | 1.27 | .15 | <.001 | [.84, 1.71] | | 1.04 | .16 | <.001 | [.59, 1.49] | | 1.02 | .14 | <.001 | [.61, 1.43] | | |  | … | | ... | | … | | … | |
| **EFT-surprise** | | | | | | | | | | | | | | | | | | | | | | | | | | | | | | | | | |  |
|  | MD | SE | *p* | [95% CI] | MD | SE | *p* | [95% CI] | | MD | SE | *p* | [95% CI] | | MD | SE | *p* | [95% CI] | | MD | SE | *p* | [95% CI] | | |  | MD | | SE | | *p* | | [95% CI] | |
| **mod-sevTBI** | … | … | … | … | … | … | … | … | | … | … | … | … | | … | … | … | … | | … | … | … | … | | |  | … | | … | | … | | … | |
| **AIS** | .38 | .24 | .619 | [-.31, 1.07] | … | … | … | … | | … | ... | … | … | | … | … | … | … | | … | … | … | … | | |  | … | | … | | … | | … | |
| **aSAH** | -.29 | .23 | .791 | [-.94, .35] | -.67 | .24 | .061 | [-1.07, .31] | | … | ... | … | … | | … | ... | … | … | | … | ... | … | … | | |  | … | | ... | | … | | … | |
| **LGG** | -.29 | .24 | .816 | [.97, .38] | -.67 | .25 | .080 | [-1.39, .04] | | -.00 | .24 | 1.000 | [-.68, .67] | | … | ... | … | … | | … | ... | … | … | | |  | … | | ... | | … | | … | |
| **PD** | -.16 | .22 | .976 | [-.78, .45] | -.54 | .23 | .180 | [1.20, .12] | | .13 | .21 | .991 | [-.48, .74] | | .13 | .23 | .992 | [-.48, .74] | | … | ... | … | … | | |  | … | | ... | | … | | … | |
| **bvFTD** | 1.10 | .22 | <.001 | [.47, .1.73] | .72 | .24 | .029 | [.05, 1.40] | | 1.39 | .22 | <.001 | [.76, 2.02] | | 1.34 | .23 | <.001 | [.73, .2.06] | | 1.26 | .21 | <.001 | [.66, 1.86] | | |  | … | | ... | | … | | … | |
| Data depicted MD= Mean difference I-II, SE= Standard Error, mod-sevTBI=moderate to severe Traumatic Brain Injury, AIS=Acute Ischemic Stroke aSAH=aneurysmal Subarachnoid Haemorrhage, LGG=Low-Grade Glioma, PD=Parkinson’s Disease, bvFTD=behavioural variant of Frontotemporal Dementia. | | | | | | | | | | | | | | | | | | | | | | | |  |  | | |  | |  | |  | |  |

|  |  |  |  |  |  |  |  |  | |  |  |  |  | |  |  |  |  | |  |  |  |  | | |  |  | |  | |  | |  | |
| --- | --- | --- | --- | --- | --- | --- | --- | --- | --- | --- | --- | --- | --- | --- | --- | --- | --- | --- | --- | --- | --- | --- | --- | --- | --- | --- | --- | --- | --- | --- | --- | --- | --- | --- |
| **Supplementary table 3** | | | | | | | | | | | | | | | | | | | | | | | |  |  | | |  | |  | |  | |  |
| Statistics post hoc ANOVA | | | | | | | | | | | | | | | | | | | | | | | |  |  | | |  | |  | |  | |  |
| **I** | **mod-sevTBI** | | | | **AIS** | | | | **aSAH** | | | | | **LGG** | | | | | **PD** | | | | |  | **bvFTD** | | | | | | | | |  |
| **DRAVLT-IR** | | | | | | | | | | | | | | | | | | | | | | | | | | | | | | | | | |  |
| **II** | MD | SE | *p* | [95% CI] | MD | SE | *p* | [95% CI] | | MD | SE | *p* | [95% CI] | | MD | SE | *p* | [95% CI] | | MD | SE | *p* | [95% CI] | | |  | MD | | SE | | *p* | | [95% CI] | |
| **mod-sevTBI** | … | … | … | … | … | … | … | … | | … | … | … | … | | … | … | … | … | | … | … | … | … | | |  | … | | … | | … | | … | |
| **AIS** | -.02 | .15 | 1.000 | [-.46, .42] | … | … | … | … | | … | ... | … | … | | … | … | … | … | | … | … | … | … | | |  | … | | … | | … | | … | |
| **aSAH** | .30 | .14 | .291 | [-.11, .71] | .32 | .15 | .279 | [-.11, .76] | | … | ... | … | … | | … | ... | … | … | | … | ... | … | … | | |  | … | | ... | | … | | … | |
| **LGG** | -.28 | .15 | .401 | [-.71, .14] | -.26 | .16 | .572 | [-.72, .19] | | -.58 | .15 | .001 | [-1.01, -.16] | | … | ... | … | … | | … | ... | … | … | | |  | … | | ... | | … | | … | |
| **PD** | -.04 | .14 | 1.000 | [-.43, .35] | -.02 | .15 | 1.000 | [-.44, .40] | | -.34 | .13 | .117 | [-.73, .04] | | .24 | .14 | .523 | [-.16, .65] | | … | ... | … | … | | |  | … | | ... | | … | | … | |
| **bvFTD** | .70 | .14 | <.001 | [.30, 1.11] | .73 | .15 | <.001 | [.29, 1.17] | | .41 | .14 | .050 | [-.00, .81] | | .99 | .15 | <.001 | [.29, 1.17] | | .75 | .14 | <.001 | [.36, 1.13] | | |  | … | | ... | | … | | … | |
| **DRAVLT-DR** | | | | | | | | | | | | | | | | | | | | | | | | | | | | | | | | | |  |
|  | MD | SE | *p* | [95% CI] | MD | SE | *p* | [95% CI] | | MD | SE | *p* | [95% CI] | | MD | SE | *p* | [95% CI] | | MD | SE | *p* | [95% CI] | | |  | MD | | SE | | *p* | | [95% CI] | |
| **mod-sevTBI** | … | … | … | … | … | … | … | … | | … | … | … | … | | … | … | … | … | | … | … | … | … | | |  | … | | … | | … | | … | |
| **AIS** | .40 | .15 | .986 | [-.34, .54] | … | … | … | … | | … | ... | … | … | | … | … | … | … | | … | … | … | … | | |  | … | | … | | … | | … | |
| **aSAH** | -.25 | .14 | .517 | [-.66, .16] | -.35 | .15 | .203 | [-.79, .09] | | … | ... | … | … | | … | ... | … | … | | … | ... | … | … | | |  | … | | ... | | … | | … | |
| **LGG** | -.19 | .15 | .806 | [-.62, .24] | -.29 | .16 | .449 | [-.75, .16] | | .06 | .15 | .999 | [-.37, .49] | | … | ... | … | … | | … | ... | … | … | | |  | … | | ... | | … | | … | |
| **PD** | -.24 | .14 | .493 | [-.63, .15] | -.34 | .15 | .182 | [-.76, .08] | | .01 | .14 | 1.000 | [-.38, .39] | | -.05 | .14 | .999 | [.46, .36] | | … | ... | … | … | | |  | … | | ... | | … | | … | |
| **bvFTD** | -.05 | .14 | .999 | [-.46, .36] | -.15 | .15 | .920 | [-.59, .29] | | .20 | .14 | .737 | [-.21, .60] | | .14 | .15 | .938 | [-.29, .57] | | .19 | .14 | .725 | [-.20, .58] | | |  | … | | ... | | … | | … | |
| **TMTA** | | | | | | | | | | | | | | | | | | | | | | | | | | | | | | | | | |  |
|  | MD | SE | *p* | [95% CI] | MD | SE | *p* | [95% CI] | | MD | SE | *p* | [95% CI] | | MD | SE | *p* | [95% CI] | | MD | SE | *p* | [95% CI] | | |  | MD | | SE | | *p* | | [95% CI] | |
| **mod-sevTBI** | … | … | … | … | … | … | … | … | | … | … | … | … | | … | … | … | … | | … | … | … | … | | |  | … | | … | | … | | … | |
| **AIS** | -.26 | .15 | .516 | [-.70, .17] | … | … | … | … | | … | ... | … | … | | … | … | … | … | | … | … | … | … | | |  | … | | … | | … | | … | |
| **aSAH** | -.61 | .14 | <.001 | [-1.02, -.21] | -.35 | .15 | .187 | [-.78, .08] | | … | ... | … | … | | … | ... | … | … | | … | ... | … | … | | |  | … | | ... | | … | | … | |
| **LGG** | -.70 | .15 | <.001 | [-1.12, -.28] | -.44 | .16 | .062 | [-.87, .01] | | -.09 | .15 | .992 | [-.51, .33] | | … | ... | … | … | | … | ... | … | … | | |  | … | | ... | | … | | … | |
| **PD** | -.07 | .13 | .994 | [-.46, .31] | .19 | .14 | .785 | [-.23, .60] | | .54 | .13 | <.001 | [.16, .92] | | .63 | .14 | <.001 | [.22, 1.03] | | … | ... | … | … | | |  | … | | ... | | … | | … | |
| **bvFTD** | .00 | .14 | 1.000 | [-.40, .41] | .27 | .15 | .494 | [-.17, .70] | | .62 | .14 | <.001 | [.22, 1.02] | | .70 | .15 | <.001 | [.28, 1.12] | | .08 | .13 | .992 | [-.31, .46] | | |  | … | | ... | | … | | … | |
| **TMTB** | | | | | | | | | | | | | | | | | | | | | | | | | | | | | | | | | |  |
|  | MD | SE | *p* | [95% CI] | MD | SE | *p* | [95% CI] | | MD | SE | *p* | [95% CI] | | MD | SE | *p* | [95% CI] | | MD | SE | *p* | [95% CI] | | |  | MD | | SE | | *p* | | [95% CI] | |
| **mod-sevTBI** | … | … | … | … | … | … | … | … | | … | … | … | … | | … | … | … | … | | … | … | … | … | | |  | … | | … | | … | | … | |
| **AIS** | .25 | .15 | .580 | [-.19, .69] | … | … | … | … | | … | ... | … | … | | … | … | … | … | | … | … | … | … | | |  | … | | … | | … | | … | |
| **aSAH** | -.28 | .14 | .377 | [-.69, .13] | -.53 | .15 | .008 | [-.96, -.09] | | … | ... | … | … | | … | ... | … | … | | … | ... | … | … | | |  | … | | ... | | … | | … | |
| **LGG** | -.21 | .15 | .682 | [-.64, .21] | -.47 | .16 | .037 | [-.92, -.02] | | .06 | .15 | .999 | [-.36, .48] | | … | ... | … | … | | … | ... | … | … | | |  | … | | ... | | … | | … | |
| **PD** | .28 | .14 | .292 | [-.10, .67] | .03 | .15 | 1.000 | [-.38, .45] | | .56 | .13 | <.001 | [.18, .95] | | .05 | .14 | .005 | [.10, .91] | | … | ... | … | … | | |  | … | | ... | | … | | … | |
| **bvFTD** | .71 | .14 | <.001 | [.30, 1.12] | .46 | .15 | .030 | [.03, .90] | | .99 | .14 | <.001 | [.59, .1.39] | | .93 | .15 | <.001 | [.51, 1.35] | | .43 | .13 | .019 | [.04. .81] | | |  | … | | ... | | … | | … | |
| Data depicted MD= Mean difference I-II, SE= Standard Error, mod-sevTBI=moderate to severe Traumatic Brain Injury, AIS=Acute Ischemic Stroke aSAH=aneurysmal Subarachnoid Haemorrhage, LGG=Low-Grade Glioma, PD=Parkinson’s Disease, bvFTD=behavioural variant of Frontotemporal Dementia, | | | | | | | | | | | | | | | | | | | | | | | |  |  | | |  | |  | |  | |  |

|  |  |  |  |  |  |  |  |  |  |  |  |  |  |  |  |  |  |  |  |  |
| --- | --- | --- | --- | --- | --- | --- | --- | --- | --- | --- | --- | --- | --- | --- | --- | --- | --- | --- | --- | --- |
| **Supplementary table 4** | | | | | | | | | | | | | | | | | | | | |
| Statistics paired sample t-tests emotion recognition | | | | | | | | | | | | | | | | | | | | |
| **mod-sevTBI** | | | | | | | | | | | | | | | | | | | | |
|  | **EFT-anger** | | | | **EFT-disgust** | | | | **EFT-fear** | | | | **EFT-sadness** | | | | **EFT-surprise** | | | |
|  | t | *p* | *d* | [95% CI] | t | *p* | *d* | [95% CI] | t | *p* | *d* | [95% CI] | t | *p* | *d* | [95% CI] | t | *p* | *d* | [95% CI] |
| **EFT-anger** | … | … | … | … | … | … | … | … | … | … | … | … | … | … | … | … | … | … | … | … |
| **EFT-disgust** | -.5 | .293 | -.05 | [-.44, .25] | … | … | … | … | … | … | … | … | … | … | … | … | … | … | … | … |
| **EFT-fear** | 1.2 | .110 | .11 | [-.10, .42] | 1.6 | .056 | .15 | [-.06, .58] | … | … | … | … | … | … | … | … | … | … | … | … |
| **EFT-sadness** | 2.3 | .012 | .21 | [.04, .59] | 2.5 | .006 | .23 | [.09, .73] | 1.2 | .123 | .11 | [-.11, .41] | … | … | … | … | … | … | … | … |
| **EFT-surprise** | -4.3 | <.001 | -.40 | [-1.22, -.45] | -3.6 | <.001 | -.33 | [-1.15, -.33] | -4.9 | <.001 | -.45 | [-1.40, -.59] | -6.1 | <.001 | -.56 | [-1.52, -.78] | … | … | … | … |
| **AIS** | | | | | | | | | | | | | | | | | | | | |
|  | t | *p* | *d* | [95% CI] | t | *p* | *d* | [95% CI] | t | *p* | *d* | [95% CI] | t | *p* | *d* | [95% CI] | t | *p* | *d* | [95% CI] |
| **EFT-anger** | … | … | … | … | … | … | … | … | … | … | … | … | … | … | … | … | … | … | … | … |
| **EFT-disgust** | 2.9 | .002 | .30 | [.15, .81] | … | … | … | … | … | … | … | … | … | … | … | … | … | … | … | … |
| **EFT-fear** | 1.9 | .031 | .20 | [-.02, .61] | -1.2 | .116 | -.13 | [-.49, .12] | … | … | … | … | … | … | … | … | … | … | … | … |
| **EFT-sadness** | 2.8 | .003 | .29 | [.14, .84] | .0 | .481 | .01 | [-.33, .35] | 1.5 | .071 | .15 | [-.07, .45] | … | … | … | … | … | … | … | … |
| **EFT-surprise** | -.9 | .198 | -.09 | [-.62, .25] | -3.2 | <.001 | -.33 | [-1.08, -.] | -2.2 | .014 | -.23 | [-.91, -.05] | -2.9 | .002 | -.31 | [-1.13, -.22] | … | … | … | … |
| **aSAH** | | | | | | | | | | | | | | | | | | | | |
|  | t | *p* | *d* | [95% CI] | t | *p* | *d* | [95% CI] | t | *p* | *d* | [95% CI] | t | *p* | *d* | [95% CI] | t | *p* | *d* | [95% CI] |
| **EFT-anger** | … | … | … | … | … | … | … | … | … | … | … | … | … | … | … | … | … | … | … | … |
| **EFT-disgust** | 2.7 | .004 | .25 | [.12, .76] | … | … | … | … | … | … | … | … | … | … | … | … | … | … | … | … |
| **EFT-fear** | 2.3 | .010 | .21 | [.05, .64] | -.7 | .231 | -.07 | [-.35, .16] | … | … | … | … | … | … | … | … | … | … | … | … |
| **EFT-sadness** | 1.8 | .039 | .16 | [-.03, .61] | -1.0 | .156 | -.09 | [-.46, .15] | -.5 | .312 | -.05 | [-.30, .18] | … | … | … | … | … | … | … | … |
| **EFT-surprise** | -2.9 | .002 | -.27 | [-.98, -.19] | -5.7 | <.001 | -.52 | [-1.38, -.67] | -5.2 | <.001 | -.48 | [-1.28, -.58] | -4.7 | <.001 | -.43 | [-1.24, -.51] | … | … | … | … |
| **LGG** | | | | | | | | | | | | | | | | | | | | |
|  | t | *p* | *d* | [95% CI] | t | *p* | *d* | [95% CI] | t | *p* | *d* | [95% CI] | t | *p* | *d* | [95% CI] | t | *p* | *d* | [95% CI] |
| **EFT-anger** | … | … | … | … | … | … | … | … | … | … | … | … | … | … | … | … | … | … | … | … |
| **EFT-disgust** | .8 | .207 | .08 | [-.20, .47] | … | … | … | … | … | … | … | … | … | … | … | … | … | … | … | … |
| **EFT-fear** | -.3 | .373 | -.03 | [-.37, .26] | -1.3 | .092 | -.13 | [-.47, .09] | … | … | … | … | … | … | … | … | … | … | … | … |
| **EFT-sadness** | 1.1 | .129 | .11 | [-.13, .47] | .2 | .420 | .02 | [-.28, .34] | 1.6 | .053 | .16 | [-.05, .49] | … | … | … | … | … | … | … | … |
| **EFT-surprise** | -5.0 | <.001 | -.50 | [-1.32, -.57] | -5.1 | <.001 | -.51 | [-.150, -.66] | -4.6 | <.001 | -.46 | [-1.28, -.50] | -5.5 | <.001 | -.55 | [-1.51, -.71] | … | … | … | … |
| **PD** | | | | | | | | | | | | | | | | | | | | |
|  | t | *p* | *d* | [95% CI] | t | *p* | *d* | [95% CI] | t | *p* | *d* | [95% CI] | t | *p* | *d* | [95% CI] | t | *p* | *d* | [95% CI] |
| **EFT-anger** | … | … | … | … | … | … | … | … | … | … | … | … | … | … | … | … | … | … | … | … |
| **EFT-disgust** | .6 | .288 | .05 | [-.21, .37] | … | … | … | … | … | … | … | … | … | … | … | … | … | … | … | … |
| **EFT-fear** | 1.5 | .073 | .12 | [-.06, .40] | .6 | .271 | .05 | [-.20, .38] | … | … | … | … | … | … | … | … | … | … | … | … |
| **EFT-sadness** | 4.1 | <.001 | .34 | [.27, .77] | 3.2 | <.001 | .26 | [.16, .71] | 3.5 | <.001 | .29 | [.15, .55] | … | … | … | … | … | … | … | … |
| **EFT-surprise** | -3.0 | .002 | -.24 | [-.80, -.16] | -3.5 | <.001 | -.29 | [-.88, -.24] | -3.8 | <.001 | -.32 | [-.98, -.32] | -6.2 | <.001 | -.52 | [-1.31, -.68] | … | … | … | … |
| **bvFTD** | | | | | | | | | | | | | | | | | | | | |
|  | t | *p* | *d* | [95% CI] | t | *p* | *d* | [95% CI] | t | *p* | *d* | [95% CI] | t | *p* | *d* | [95% CI] | t | *p* | *d* | [95% CI] |
| **EFT-anger** | … | … | … | … | … | … | … | … | … | … | … | … | … | … | … | … | … | … | … | … |
| **EFT-disgust** | 2.0 | .024 | .18 | [.00, .49] | … | … | … | … | … | … | … | … | … | … | … | … | … | … | … | … |
| **EFT-fear** | -.3 | .384 | -.03 | [-.27, .20] | -2.5 | .007 | -.22 | [-.51, -.06] | … | … | … | … | … | … | … | … | … | … | … | … |
| **EFT-sadness** | 3.4 | <.001 | .30 | [.17, .65] | 1.4 | .077 | .13 | [-.06, .39] | 3.9 | <.001 | .34 | [.22, .67] | … | … | … | … | … | … | … | … |
| **EFT-surprise** | -2.3 | .012 | -.20 | [-.64, -.05] | -4.0 | <.001 | -.35 | [-.88, -.30] | -1.8 | .036 | -.16 | [-.64, .03] | -4.6 | <.001 | -.40 | [-1.08, -.43] | … | … | … | … |
| Data depicted mod-sevTBI=moderate to severe Traumatic Brain Injury, AIS=Acute Ischemic Stroke aSAH=aneurysmal Subarachnoid Haemorrhage, LGG=Low-Grade Glioma, PD=Parkinson’s Disease, bvFTD=behavioural variant of Frontotemporal Dementia, | | | | | | | | | | | | | | | | | | | | |

|  |  |  |  |  |  |  |  |  |  |  |  |  |  |  |  |  |  |  |  |  |
| --- | --- | --- | --- | --- | --- | --- | --- | --- | --- | --- | --- | --- | --- | --- | --- | --- | --- | --- | --- | --- |
| **Supplementary table 5** | | | | | | | | | | | | | | | | | | | | |
| Statistics paired sample t-tests general cognition | | | | | | | | | | | | | | | | | | | | |
| **mod-sevTBI** | | | | | | | | | | | | | | | | | | | | |
|  | **EFT-total** | | | | **DRAVLT-IR** | | | | **DRAVLT-DR** | | | | **TMTA** | | | | **TMTB** | | | |
|  | t | *p* | *d* | [95% CI] | t | *p* | *d* | [95% CI] | t | *p* | *d* | [95% CI] | t | *p* | *d* | [95% CI] | t | *p* | *d* | [95% CI] |
| **EFT-total** | … | … | … | … | … | … | … | … | … | … | … | … | … | … | … | … | … | … | … | … |
| **DRAVLT-IR** | -.8 | .210 | -.08 | [-.36, .15] | … | … | … | … | … | … | … | … | … | … | … | … | … | … | … | … |
| **DRAVLT-DR** | -4.6 | <.001 | -.43 | [-.96, -.38] | -4.2 | <.001 | -.39 | [-.84, -.30] | … | … | … | … | … | … | … | … | … | … | … | … |
| **TMTA** | -1.0 | .162 | -.09 | [-.38, .13] | .3 | .373 | .03 | [-.23, .32] | -3.7 | <.001 | -.35 | [-.81, -.24] | … | … | … | … | … | … | … | … |
| **TMTB** | -4.1 | <.001 | -.38 | [-.73, -.26] | 3.1 | .001 | .29 | [.15, .67] | -1.0 | .149 | -.01 | [-.43, .13] | -3.6 | <.001 | -.34 | [-.57, -.17] | … | … | … | … |
| **AIS** | | | | | | | | | | | | | | | | | | | | |
|  | t | *p* | *d* | [95% CI] | t | *p* | *d* | [95% CI] | t | *p* | *d* | [95% CI] | t | *p* | *d* | [95% CI] | t | *p* | *d* | [95% CI] |
| **EFT-total** | … | … | … | … | … | … | … | … | … | … | … | … | … | … | … | … | … | … | … | … |
| **DRAVLT-IR** | -.7 | .237 | -.08 | [-.30, .14] | … | … | … | … | … | … | … | … | … | … | … | … | … | … | … | … |
| **DRAVLT-DR** | -4.1 | <.001 | -.43 | [-.77, -.27] | -3.1 | .001 | -.33 | [-.71, -.16] | … | … | … | … | … | … | … | … | … | … | … | … |
| **TMTA** | -2.5 | .007 | -.27 | [-.60, -.07] | 1.6 | .058 | .17 | [-.06, .53] | -1.4 | .076 | -.16 | [-.54, .09] | … | … | … | … | … | … | … | … |
| **TMTB** | -1.4 | .080 | -.15 | [-.45, .08] | .7 | .258 | .07 | [-.20, .39] | -2.1 | .021 | -.22 | [-.67, -.01] | 1.6 | .058 | .17 | [-.04, .32] | … | … | … | … |
| **aSAH** | | | | | | | | | | | | | | | | | | | | |
|  | t | *p* | *d* | [95% CI] | t | *p* | *d* | [95% CI] | t | *p* | *d* | [95% CI] | t | *p* | *d* | [95% CI] | t | *p* | *d* | [95% CI] |
| **EFT-total** | … | … | … | … | … | … | … | … | … | … | … | … | … | … | … | … | … | … | … | … |
| **DRAVLT-IR** | 6.0 | <.001 | .55 | [.49, .96] | … | … | … | … | … | … | … | … | … | … | … | … | … | … | … | … |
| **DRAVLT-DR** | -2.9 | .002 | .26 | [-.65, -.12] | -8.0 | <.001 | -.74 | [-1.38, -.84] | … | … | … | … | … | … | … | … | … | … | … | … |
| **TMTA** | -1.9 | .030 | -.17 | [-.48, .01] | 7.4 | <.001 | .68 | [.71, 1.22] | -1.0 | .166 | -.09 | [-.40, .14] | … | … | … | … | … | … | … | … |
| **TMTB** | -2.4 | .009 | -.22 | [-.52, -.05] | 8.2 | <.001 | .75 | [.76, 1.24] | -.6 | .260 | -.06 | [-.39, .20] | -.5 | .326 | -.04 | [-.24, .15] | … | … | … | … |
| **LGG** | | | | | | | | | | | | | | | | | | | | |
|  | t | *p* | *d* | [95% CI] | t | *p* | *d* | [95% CI] | t | *p* | *d* | [95% CI] | t | *p* | *d* | [95% CI] | t | *p* | *d* | [95% CI] |
| **EFT-total** | … | … | … | … | … | … | … | … | … | … | … | … | … | … | … | … | … | … | … | … |
| **DRAVLT-IR** | -.4 | .357 | -.04 | [-.30, .20] | … | … | … | … | … | … | … | … | … | … | … | … | … | … | … | … |
| **DRAVLT-DR** | -3.7 | <.001 | -.37 | [-.79, -.24] | -3.4 | <.001 | -.34 | [-.74, -.19] | … | … | … | … | … | … | … | … | … | … | … | … |
| **TMTA** | -3.7 | <.001 | -.37 | [-.77, -.23] | 3.3 | <.001 | .33 | [.18, .72] | -.2 | .435 | -.02 | [-.24, .20] | … | … | … | … | … | … | … | … |
| **TMTB** | -2.6 | .005 | -.26 | [-.68, -.10] | 2.6 | .006 | .26 | [.08, .62] | -.9 | .187 | -.09 | [-.38, .14] | 1.2 | .116 | .12 | [-.07, .30] | … | … | … | … |
| **PD** | | | | | | | | | | | | | | | | | | | | |
|  | t | *p* | *d* | [95% CI] | t | *p* | *d* | [95% CI] | t | *p* | *d* | [95% CI] | t | *p* | *d* | [95% CI] | t | *p* | *d* | [95% CI] |
| **EFT-total** | … | … | … | … | … | … | … | … | … | … | … | … | … | … | … | … | … | … | … | … |
| **DRAVLT-IR** | 3.9 | <.001 | .32 | [.22, .66] | … | … | … | … | … | … | … | … | … | … | … | … | … | … | … | … |
| **DRAVLT-DR** | -2.6 | .006 | -.21 | [-.57, -.07] | -5.7 | <.001 | -.47 | [-1.03, -.50] | … | … | … | … | … | … | … | … | … | … | … | … |
| **TMTA** | 3.4 | <.001 | .29 | [.16, .60] | .5 | .308 | .04 | [-.15, .26] | -5.2 | <.001 | -.43 | [-.97, -.44] | … | … | … | … | … | … | … | … |
| **TMTB** | 3.0 | .001 | .25 | [.13, .62] | .5 | .307 | .04 | [-.15, .26] | -5.2 | <.001 | -.43 | [-.97, -.43] | .0 | .500 | .00 | [-.17, -.17] | … | … | … | … |
| **bvFTD** | | | | | | | | | | | | | | | | | | | | |
|  | t | *p* | *d* | [95% CI] | t | *p* | *d* | [95% CI] | t | *p* | *d* | [95% CI] | t | *p* | *d* | [95% CI] | t | *p* | *d* | [95% CI] |
| **EFT-total** | … | … | … | … | … | … | … | … | … | … | … | … | … | … | … | … | … | … | … | … |
| **DRAVLT-IR** | -1.7 | .049 | -.15 | [-.37, -.03] | … | … | … | … | … | … | … | … | … | … | … | … | … | … | … | … |
| **DRAVLT-DR** | -11.8 | <.001 | -1.08 | [-1.74, -1.24] | -9.2 | <.001 | -.84 | [-1.60, -1.03] | … | … | … | … | … | … | … | … | … | … | … | … |
| **TMTA** | -8.5 | <.001 | -.78 | [-1.11, -.69] | 6.0 | <.001 | .55 | [.49, .98] | -3.7 | <.001 | -.34 | [-.91, -.27] | … | … | … | … | … | … | … | … |
| **TMTB** | -5.0 | <.001 | -.46 | [-.78, -.34] | 3.4 | <.001 | .31 | [.16, .63] | -5.9 | <.001 | -.54 | [-1.24, -.61] | 4.1 | <.001 | .38 | [.18, .50] | … | … | … | … |
| mod-sevTBI=moderate to severe Traumatic Brain Injury, AIS=Acute Ischemic Stroke aSAH=aneurysmal Subarachnoid Haemorrhage, LGG=Low-Grade Glioma, PD=Parkinson’s Disease, bvFTD=behavioural variant of Frontotemporal Dementia, | | | | | | | | | | | | | | | | | | | | |
